# Technological breakthroughs and advancements in the application of mRNA vaccines: a comprehensive exploration and future prospects

**DOI:** 10.3389/fimmu.2025.1524317

**Published:** 2025-03-04

**Authors:** Zhimeng Wei, Shuai Zhang, Xingya Wang, Ying Xue, Sheng Dang, Jingbo Zhai

**Affiliations:** ^1^ School of Basic Medical Sciences, Inner Mongolia Minzu University, Tongliao, China; ^2^ Keerqin District First People’s Hospital, Tongliao, China; ^3^ Brucellosis Prevention and Treatment Engineering Research Center of Inner Mongolia Autonomous Region, Tongliao, China; ^4^ Key Laboratory of Zoonose Prevention and Control at Universities of Inner Mongolia Autonomous Region, Tongliao, China

**Keywords:** mRNA vaccines, immunogenicity, classification, design, delivery vector development, stability, biomedical application

## Abstract

mRNA vaccines utilize single-stranded linear DNA as a template for *in vitro* transcription. The mRNA is introduced into the cytoplasm via the corresponding delivery system to express the target protein, which then performs its relevant biological function. mRNA vaccines are beneficial in various fields, including cancer vaccines, infectious disease vaccines, protein replacement therapy, and treatment of rare diseases. They offer advantages such as a simple manufacturing process, a quick development cycle, and ease of industrialization. Additionally, mRNA vaccines afford flexibility in adjusting antigen designs and combining sequences of multiple variants, thereby addressing the issue of frequent mutations in pathogenic microorganisms. This paper aims to provide an extensive review of the global development and current research status of mRNA vaccines, with a focus on immunogenicity, classification, design, delivery vector development, stability, and biomedical application. Moreover, the study highlights current challenges and offers insights into future directions for development.

## 1 Introduction

Vaccination is the most cost-effective strategy for controlling the spread of infectious diseases, significantly contributing to disease prevention and control in both humans and animals (1, 2). As a result of extensive vaccine use, viruses like smallpox and cowpox have been completely eradicated worldwide (1). Traditional vaccine development follows a mature model of isolation, attenuation/inactivation, and injection; the body elicits a protective immune response by recognizing attenuated or inactivated pathogens (3, 4). However, the technological production process of traditional vaccines is intricate, with a lengthy development cycle. This poses challenges with regard to industrialization and applicability to emerging infectious diseases and cancer (5).

Unlike traditional vaccines, mRNA vaccines bypass these challenges. Their potential as promising alternative strategies to traditional vaccines has gained widespread attention. This is due to their simple production process, short development cycle, ease of industrialization, adaptability to new variations, and their capacity to evoke a strong immune response (2, 6). Amid the coronavirus disease 2019 (COVID-19) pandemic, BNT162b2 (Pfizer-BioNTech) and mRNA-1273 (Moderna) were introduced and received US FDA approval, marking the successful clinical application of mRNA vaccines. The humoral and cellular immunity stimulated by these mRNA vaccines are significantly higher than traditional vaccines, with over 90% protection efficiency against infection by the severe acute respiratory syndrome coronavirus 2 (SARS-CoV-2) (6–9).

On March 22, 2023, the SARS-CoV-2 mRNA vaccine SYS6006 was introduced in China, marking the first domestically developed and available mRNA COVID-19 vaccine (9). As early as 1990, scientists injected mRNA containing the target gene, prepared *in vitro*, into mice and discovered that the target gene expressed the corresponding protein, generating an immune response (10). Based on this discovery, researchers proposed constructing mRNA containing the target gene and introducing it into the body through a delivery system. Such a target gene can express antigens related to certain infectious diseases or tumors, stimulating the body’s humoral and cellular immunity and thus potentially preventing and treating infectious diseases and cancer (11).

mRNA vaccines do not pose any risk of infection and offer several advantages compared to other types of vaccines. These include: a. stronger safety guarantees as the translation of mRNAs occurs in the cytoplasm without entering the nucleus, thereby eliminating concerns about mRNA integration into the host genome (12); b. an enhanced ability to address the unsolved problems of traditional vaccines, including improved safety and efficacy. such as live attenuated vaccines, which may exhibit weak toxicity and a potential risk for virulence reversion, limiting their application among pregnant women and immuno-compromised individuals; inactivated vaccines and recombinant protein vaccines have poor immunogenicity and need to be co-delivered with adjuvants to induce sufficient immune responses. Moreover, the host’s immune cells usually can only recognize them as exogenous antigens. After being endocytosed by antigen-presenting cells (APCs), they are presented through Major Histocompatibility Complex (MHC)-II and can only effectively activate CD4^+^ T cells, thereby activating humoral immunity but having no cellular immune effect; compared to the above vaccines (1, 13–15); c. reduced production costs and the avoidance of contamination from viral, bacterial, or cellular impurities, making large-scale batch production possible, while the manufacturing of inactivated vaccines and live attenuated vaccines typically involves cell cultivation. Due to the different pathogens, diverse cultivation environments and conditions are required. These factors constrain the technology transfer, leading to a decrease in globalize manufacturing production (8, 16); d. the flexibility of modifying mRNA molecules and selecting appropriate delivery routes, which can enhance stability, extend half-life, improving translation efficiency, and increase immunogenicity can enable mRNA to exert its maximum efficacy (17).

The antigens translated *in situ* by mRNA vaccines closely resemble the authentic viral antigens. Because after mRNA vaccination, the process of using raw materials within cells to product target antigens is akin to the production of antigens expressed by viruses that invade the organism. Therefore, both exhibit a high degree of consistency in spatial conformation and modification, eliciting robust humoral and cellular immune responses (18). mRNA vaccine technology is a promising platform with rapid, scalable features, highlighting significant therapeutic potential for the treatment of various diseases (19). This article provides a comprehensive review of immunogenicity, classification, design, delivery vector development, stability, biomedical application, challenges, and future developments of mRNA vaccines. It serves as a reference for research and development in fields such as infectious diseases, cancer, protein replacement therapy, and the treatment of rare diseases.

## 2 mRNA vaccines immunogenicity

mRNA vaccines induce both innate and adaptive immune responses by introducing mRNA, containing the target gene, into cells using a delivery vector, subsequently translating it into the target protein. They can be administered in various ways (intramuscularly, intracutaneously, or subcutaneously), as these methods enable transfection into three types of host cells: non-immune cells at the injection site (muscle cells and epidermal cells); immune cells at the injection site (dendritic cells and macrophages); and immune cells in peripheral lymphoid organs, after injection, as the mRNA is transferred through the lymphatic system to the adjacent lymph nodes (LNs) or spleen (2).

The encapsulated mRNA enters the cells via endocytosis, forming endosomes within the cells. The mRNA is then released from these endosomes and enters the cytoplasm. Here, it is translated into target proteins by ribosomes. As an endogenous antigen, the expressed protein undergoes proteasome processing to create antigenic peptides. These peptides bind to MHC-I molecules and are presented to CD8^+^ T cells, thereby activating cellular immunity (20). The expressed protein can also be secreted into the extracellular environment, forming an exogenous antigen. Upon entering the circulatory system, B-cell receptors on the surface of B cells recognize this protein. Simultaneously, B cells form endosomes containing the exogenous antigen by phagocytosis, which then fuse with lysosomes. These create an endosome/lysosome hybrid organelle where the exogenous antigen is degraded into antigenic peptides by proteases. As APCs, B cells can bind these antigenic peptides to MHC class II molecules, subsequently activating CD4^+^ T cells. Proteins secreted into the circulatory system also get consumed by other APCs. These APCs process exogenous antigens, thus allowing the binding of antigenic peptides to MHC-II molecules and presentation to CD4^+^ T cells. Once activated, CD4^+^ T cells release cytokines that enhance cellular immunity and aid B cells in antibody production (21).

As a Pathogen-Associated Molecular Pattern (PAMP), mRNA binds to Pattern Recognition Receptors (PRRs) on the surface of innate immune cells, thereby activating innate immune responses. Single-stranded RNA (ssRNA) activates Toll-like Receptors (TLRs) such as TLR7 and TLR8, leading to the activation of the Myeloid Differentiation Primary Response 88 (MyD88) pathway. Double-stranded RNA (dsRNA) activates multiple sensors, including TLR3, Retinoic acid-inducible gene I (RIG-I), Melanoma Differentiation-Associated protein 5 (MDA-5), Protein Kinase R (PKR), and Oligoadenylate Synthetase (OAS). Among these, TLR3, RIG-I, and MDA-5 initiate downstream signaling pathways through TIR domain-containing adaptor-inducing IFN-β (TRIF) and mitochondrial antiviral signaling (MAVS) proteins. MyD88, TRIF, and MAVS pathways encourage the production of type I interferon (IFN-I) and pro-inflammatory cytokines. IFN-I not only enhances the immune system but also activates PKR and OAS, exerting an inhibitory effect on mRNA replication (2, 22–24).

Upon PKR activation, eukaryotic translation initiation factor 2 (eIF2) undergoes phosphorylation which blocks mRNA translation. Additionally, dsRNA binding to OAS activates Ribonuclease L (RNase L), leading to the degradation of exogenous RNA. Besides dsRNA impurities, improperly designed mRNA structures could also activate PRRs such as MDA-5 and PKR, resulting in inhibited antigen expression. Therefore, improving the purification process and optimizing the *in vitro* transcription (IVT) mRNA design is vital (14).

**Figure 1:**
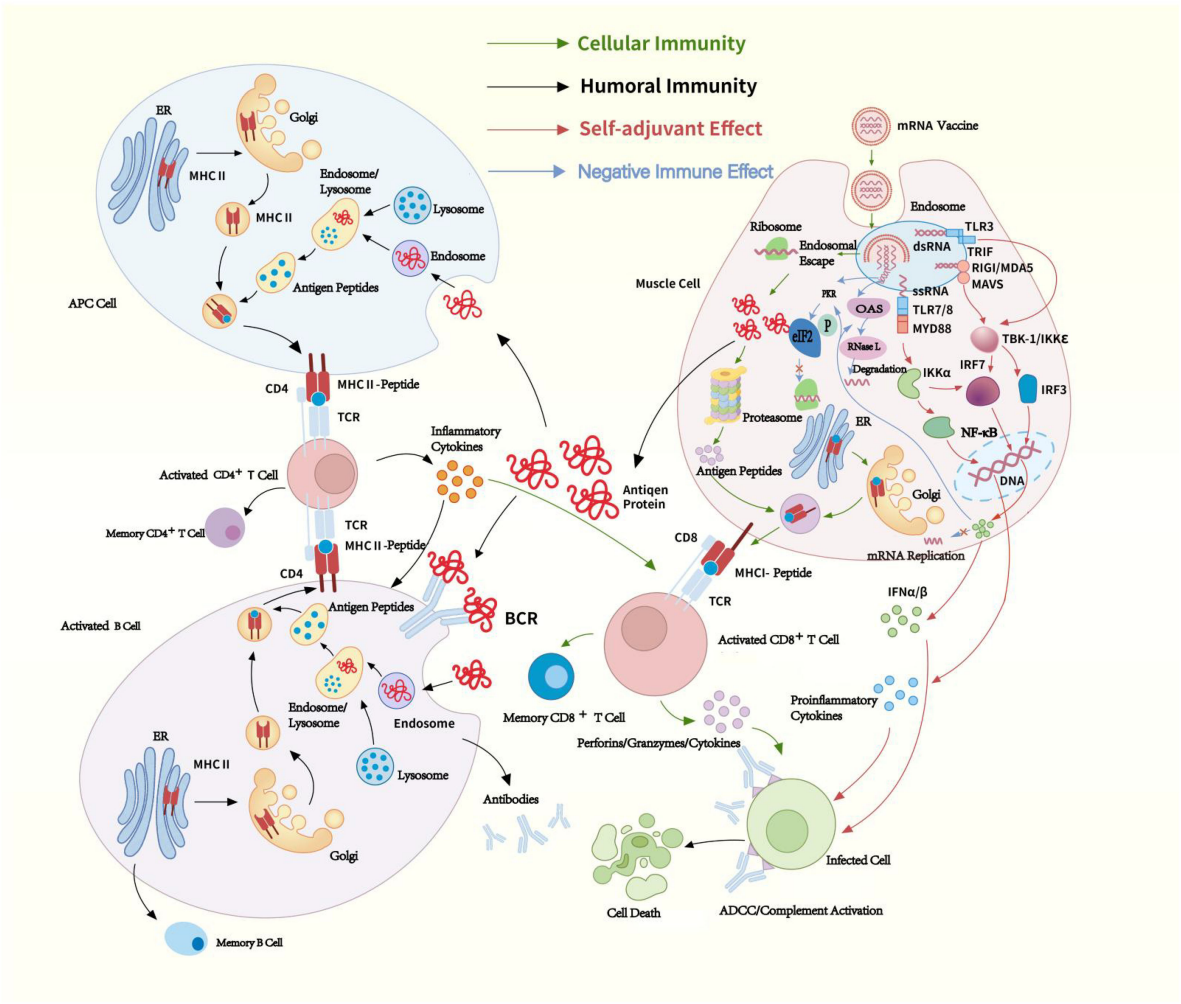
mRNA vaccines immunogenicity. The mRNA delivered in mRNA vaccines enters cells through endocytosis and is subsequently released from endosome into the cytoplasm, where ribosomes translate it into proteins. The proteins are degraded into peptides by proteasomes and presented on the cell surface as antigens by MHC-I. These antigens bind to T-cell receptors (TCRs), activating CD8^+^ T cells, which secrete perforins and granzymes to kill infected cells. Proteins secreted extracellularly are phagocytosed by APCs and degraded into peptides within lysosomes. These peptides are then presented on the cell surface by MHC-II for recognition by CD4^+^ T cells. CD4^+^ T cells can not only activate cellular immune responses by secreting cytokines but also induce humoral immune responses by activating B cells. ssRNA in mRNA vaccines binds to Toll-like receptor (TLR) 7/8 within endosomes, while dsRNA interacts with TLR3, Retinoic acid-inducible gene I (RIG-I), and Melanoma Differentiation-Associated protein 5 (MDA-5). These interactions induce the production of pro-inflammatory cytokines and type I IFN (IFN-I), thereby activating antiviral innate immune responses, through which mRNA exhibits a self-adjuvant effect. Additionally, dsRNA also can activate PKR and OAS. IFN-I binds to Interferon-α/β Receptor (IFNAR) and trigger the JAK-STAT signaling pathway, inducing the expression of PKR and OAS, thereby exerting a negative regulatory effect on the immune response. Green arrow: cellular immunity, black arrow: homoral immunity, red arrow: self-adjuvant effect, blue arrow: negative immune effect. TCR, T-cell receptor; BCR, B-cell receptor; MHC, Major Histocompatibility Complex; APC, antigen-presenting cell; ADCC, antibody-dependent cellular cytotoxicity; RNase L, Ribonuclease L; eIF2, eukaryotic translation initiation factor 2; dsRNA, Double-stranded RNA; ssRNA, Single-stranded RNA; TBK-1, TANK-binding kinase 1; IKKϵ, IκB kinase ϵ; IFN, interferon; IRF, IFN regulatory factor; MyD88, Myeloid Differentiation Primary Response 88; ER, Endoplasmic Reticulum.

Given that mRNA can activate downstream interferon-related pathways, IFN-I propels the maturation and activation of APCs. This promotes antigen presentation and triggers a powerful adaptive immune response, acting like an adjuvant effect (**Figure 1**).

## 3 mRNA vaccines classification

mRNA vaccines are largely categorized into four types: non-replicating mRNA (nrmRNA) vaccines, self-amplifying RNA (saRNA) vaccines, trans-amplifying RNA (taRNA) vaccines, and circular RNA (circRNA) vaccines (25, 26). nrmRNA vaccines deliver genetic information encoding a specific antigen but do not self-replicate (6). The intensity of the immune response is proportionate to the transcript amount. Due to its non-amplifying nature, high doses of mRNA may be necessary, potentially leading to repeated mRNA administration (27).

saRNA is significantly larger than nrmRNA (approximately 9-12 kb). saRNA shares similar features with nrmRNA, such as a 5’ Cap, a 3’ poly(A), and 5’ and 3’ untranslated regions (UTRs). Additionally, saRNA contains a large open reading frame (ORF), four non-structural proteins (nsP1-4), and a subgenomic promoter (SGP) (28). The saRNA vaccine mimics the replication characteristics of alphaviruses, replacing the gene encoding viral structural proteins, typically located after the SGP in the viral genome, with a heterologous gene encoding the desired protein. nsP1 is a membrane-anchoring protein that mediates the binding of the replicase complex to the plasma membrane, causing saRNA to replicate in membrane invaginations. Additionally, it functions as a capping enzyme, responsible for capping the 5’ end of the positive-strand RNA. nsP2 has RNA triphosphatase, NTPase, helicase, and protease functions, supporting RNA replication and polyprotein processing. The function of nsP3 is not yet fully understood, but it has been established that nsP3 interacts with host proteins, which either promote or inhibit RNA replication. nsP4 is an RNA-dependent RNA polymerase (RDRP) that forms the core activity responsible for intracellular RNA amplification. Additionally, through its terminal adenylyltransferase activity, nsP4 synthesizes new positive-strand RNA poly(A) tails. The nsP1–4 work together to form a replicase complex. saRNA also holds conserved sequence elements (CSEs) from the alphavirus genome. Among them, the first CSE is located within the 5’-UTR, the second is at the 5’ end of the nsP1 ORF, the third is located at the start site of the subgenomic RNA (SGP), and the fourth is at the 3’ end. CSEs and SGP, as promoters of the replicase complex, allow the replicase complex to selectively dock on saRNA and initiate full-length genomic and subgenomic RNA-dependent RNA transcription (2, 28–30). SaRNA vaccines offer key advantages such as lower dosage requirements and activation of the host’s immune system. During its replication, saRNA generates dsRNA, which acts as an adjuvant by stimulating the host’s innate immune response (30). However, this response can be a “ double-edged sword “. While moderate activation enhances vaccine efficacy, excessive activation may inhibit saRNA expression and induce an excessive inflammatory response, ultimately undermining the effectiveness of the saRNA vaccine (6). While nucleoside modifications are effective in reducing immunogenicity, they cannot be applied to saRNA (27). Additionally, the replicase and the antigen-encoding gene of saRNA vaccines are both located on the same mRNA molecule, resulting in a longer RNA sequence, which present significant challenges in terms of production, delivery, and stability (30).

Based on saRNA technology, taRNA vaccines separate the transreplicon (TR) sequences, which encode the target protein, and the nsP1-4 sequences. These are carried on two separate linear mRNA molecules. It’s translation efficiency is significantly higher than saRNA vaccines (29). The replicase complex of taRNA is produced by translating nrmRNA encoded for replication. However, without CSEs, it cannot be amplified through replication. In contrast, the TR contains CSEs and when the replicase complex binds, it triggers trans-replication (30).

**Figure 2:**
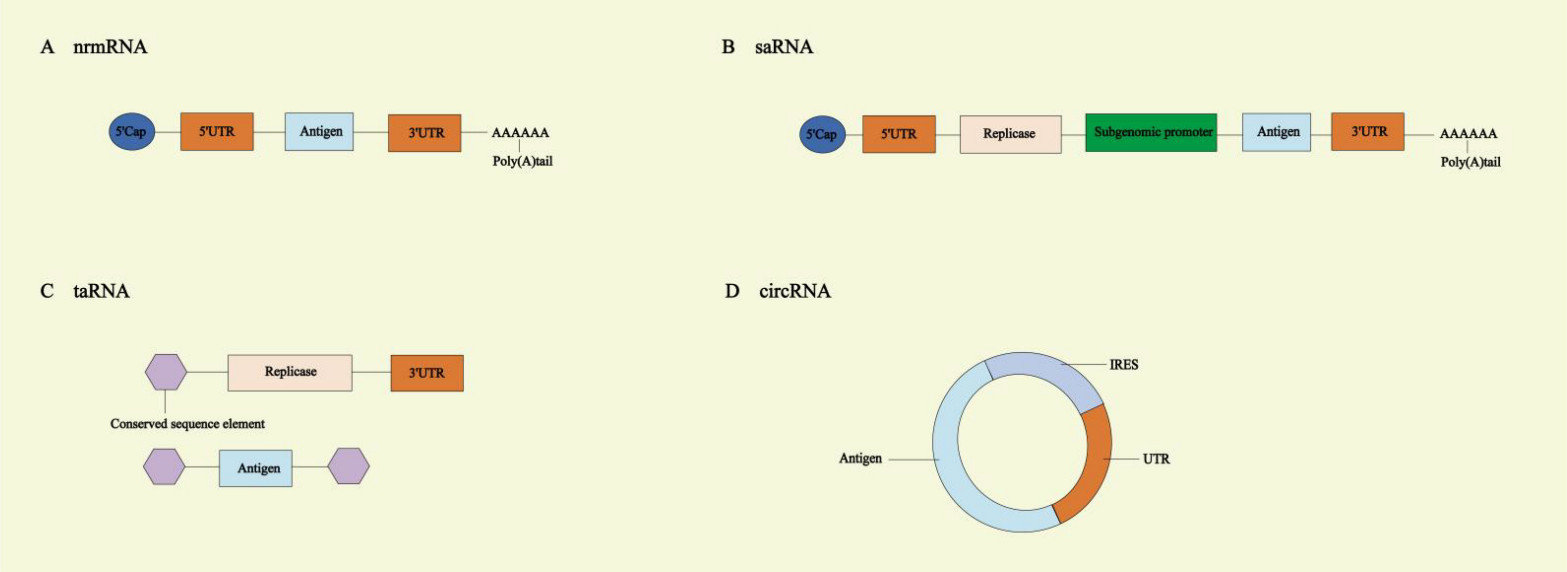
Different types of mRNA vaccines. **(A)** Non-replicating mRNA (nrmRNA) vaccines. **(B)** Self-amplifying RNA (saRNA) vaccines. **(C)** Trans-amplifying RNA (taRNA) vaccines. **(D)** Circular RNA (circRNA) vaccines. UTR, untranslated region; IRES, internal ribosome entry site.

The circRNA molecule is a single-stranded, covalently closed circular RNA structure, and lacks a 5’cap structure and 3’ poly-A tail. It is unaffected by RNA exonucleases and exhibits higher stability compared to linear RNA (31, 32). CircRNA offers several advantages compared to unmodified nrmRNA, triggering fewer adverse immune responses and generating a higher amount of neutralizing antibodies (33). It can continue to express in cells for 5 to 7 days, while linear mRNA with the same sequence lasts only 2 days (34). CircRNA vaccines lack a cap structure, but translation can be driven by adding internal ribosome entry site (IRES) elements to the 5’UTR. The IRES interacts with initiation factors, such as eIF4G2 or the eIF3 complex, to recruit the 40S ribosomal subunit, thereby driving translation. Its activity is regulated by IRES trans-acting factors (ITAFs) (2, 35). During the design of circRNA, UTRs containing RNA-binding protein (RBP) motifs can be introduced, allowing RBPs to facilitate translation initiation. CircRNA synthesis methods include chemical ligation, enzymatic ligation, and ribozyme-mediated ligation. In ribozyme ligation, the introduction of homology arms and unstructured spacer sequences promotes circularization, which can further enhance translation efficiency (36–38) (**Figure 2**).

## 4 mRNA design

### 4.1 mRNA vaccine structural elements

Mature eukaryotic mRNAs contain five elements: a 5’ cap, the 5’ UTR, an ORF, a 3’ UTR, and a 3’ polyadenylated tail (3’ Poly (A)). The therapeutic efficacy of mRNA vaccines is limited by factors like their short half-life and low protein expression levels. Therefore, through chemical modifications and optimized sequence design of these five elements, we can improve the stability, immunogenicity, and translational efficiency (39).

### 4.2 mRNA vaccine processing and modification

#### 4.2.1 5’ cap structure

The 5’ cap is a special structure located at the 5’ end of mRNA, and it is a conserved modification that occurs during the transcription process of eukaryotic mRNA (40). mRNAs created by IVT mimic those of eukaryotic organisms. The 7-methylguanosine is linked to the first nucleotide at the 5’ end of the mRNA via a triphosphate bond in a reverse 5’-5’ manner, forming the m^7^G cap structure, also known as Cap 0 (m^7^GpppNp) (41). The cap structure is essential for mRNA translation initiation. The eIF4E assists in the recognition and binding of the mRNA by the ribosomal small subunit, which allows translation to begin from AUG and regulates the smooth progression of protein translation (42, 43).

The 5’ cap shields the 5’ end of the mRNA, protecting it from 5’→3’ exonucleases, enhancing stability, improving translation efficiency, and reducing the immunogenicity of mRNA (43). This cap plays a role in mRNA precursor splicing, nuclear export, subcellular localization, and decay (43–47). The RIG-I is a PRR induced by retinoic acid (vitamin A), which binds to the 5’-diphosphate or 5’-triphosphate end and the short blunt double-stranded portion of uncapped viral RNA. This binding activates innate immune cells to release IFN-α/β and trigger an antiviral immune response (48–51). The presence of the cap structure on mRNA prevents RIG-I from recognizing and binding to it, thereby helping the body distinguish between exogenous triphosphorylated viral RNA and endogenous RNA (52).

The 5’ cap forms through the concerted catalytic action of RNA triphosphatase (RTPase), guanylyltransferase (GTase), guanine-N7-methyltransferase (N7MTase), and 2’-O-methyltransferase (2’-O-MTase). Based on the degree of methylation, three types of cap structures can be formed: Cap 0, Cap 1, and Cap 2. In higher eukaryotic cells, Cap 1 is the dominant cap structure. The Cap 0 structure is the most basic (m^7^GpppNp); however, mRNA containing Cap 0 is easily recognized by the host as exogenous RNA, which can activate the innate immune system and trigger an inflammatory response (2, 53). The Cap 1 structure (m^7^GpppN1mp) involves the 2’-O-methylation of the first nucleotide at the 5’ end of mRNA linked to the cap. Since Cap 1 has, so far, only been described in eukaryotic cell mRNA, it can serve as a self-RNA marker, reducing RIG-I activation and increasing mRNA translation efficiency *in vivo* (2).

The degree of mRNA methylation affects its binding to RIG-I. RIG-I can bind to Cap 0 or Cap 1 mRNA, but the binding to Cap 2 is significantly reduced. Cap 2 (m^7^GpppN1mpN2mp) is a structure formed when the second nucleotide on the mRNA’s 5’ end undergoes 2’-O-methylation through cap methyltransferase 2 (CMTR2) acting on Cap 1 mRNA, leading to 2’-O-methylation of both the first and second nucleotides at the mRNA 5’ end. This Cap 2 form inhibits the activation of RIG-I, thereby enhancing mRNA translation efficiency (54). Currently, the Cap 1 structure is the most common capping method for mRNA vaccines (2). Alongside natural cap structures, various cap analogs are often used during the mRNA IVT process to enhance the structural stability of mRNA. Examples include anti-reverse cap analogs (ARCAs) and Cap 1-like structures. There are two methods of capping during the mRNA IVT process: enzyme capping and co-transcriptional capping.

##### 4.2.1.1 Enzyme capping method

**Figure 3:**
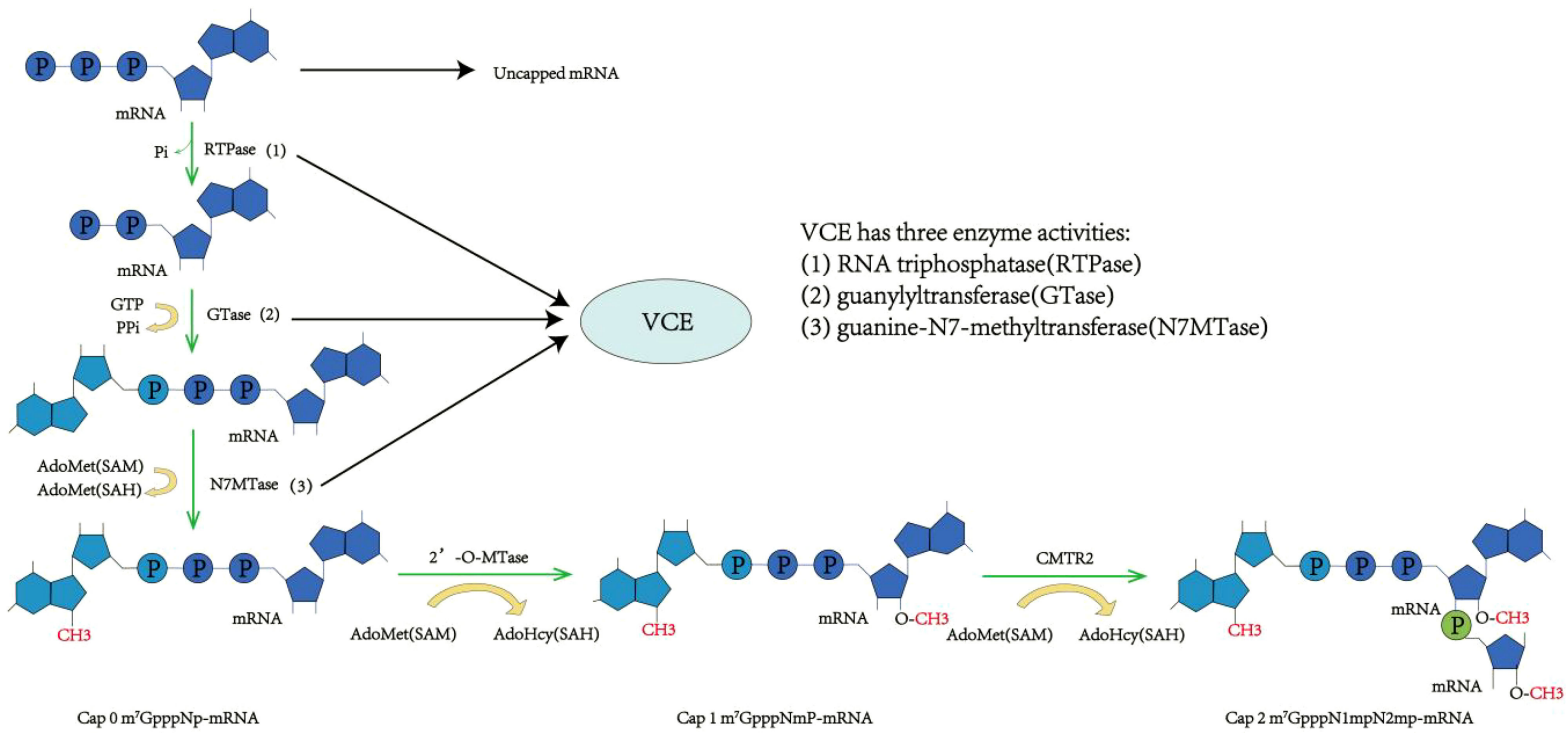
Enzyme capping method. RNA triphosphatase (RTPase) hydrolyzes the 5’γ-phosphate of messenger RNA (mRNA), resulting in a β-phosphate group. Under the action of guanylyltransferase (GTase), the β-phosphate at the 5’ end is linked to guanosine monophosphate (GMP) via a 5’-to-5’ triphosphate bridge. RNA guanine-N7 methyltransferase (G-N7 MTase) uses S-adenosyl-L-methionine (AdoMet) as a substrate to methylate the guanine base at the N7 position, forming Cap 0. Subsequently, 2’-O-methyltransferase methylates the R1 group on Cap 0 to produce Cap 1. Further methylation at the R2 position results in the formation of Cap 2.

This method of enzymatic capping mimics the capping process in eukaryotic cells, using capping enzymes for transcription before capping. This process is made up of four steps: a. hydrolysis of the γ-phosphate at the mRNA 5’ end by RTPase, resulting in mRNA 5’ β-phosphate formation; b. the linking of the GMP moiety in GTP to the mRNA 5’ end β-phosphate using GTase, forming guanosine triphosphate (40, 43); c. N7MTase-mediated methylation of the N7 position of the mRNA 5’ end guanine base with S-adenosylmethionine (SAM) as the methyl donor, resulting in Cap 0 formation (m^7^GpppNp); d. methylation of the 2’-O position of the first nucleotide at the mRNA 5’ end to form Cap 1 (m^7^GpppN1mp) and of both the first and second nucleotides at the 2’-O position to form Cap 2 (m^7^GpppN1mpN2mp), both under the activity of 2’-O-MTase using SAM as the methyl donor (40). The Vaccinia capping enzyme (VCE) from the cowpox virus has the three necessary enzyme activities for Cap 0 formation, including RTPase, GTase, and N7MTase (55, 56). Thereby, Cap 0 can be directly established using VCE with IVT mRNA, NTPs, SAM, and other substrates. Then, under the influence of 2’-O-MTase, Cap 1 can be developed further (**Figure 3**) (55, 57). Moderna’s mRNA-1273 vaccine uses VCE for capping, achieving a capping rate of 100% (58). The enzyme-based capping method, which involves transcription before capping, brings in additional proteins and SAM, creating a complex process that requires several purifications, enhances quality control, and necessitates precision temperature control, thus complicating the scale-up of the procedure.

##### 4.2.1.2 Co-transcriptional capping method

In the IVT reaction system, cap analogs and bacteriophage RNA polymerases (T7, T3, or Sp6) are added to achieve mRNA co-transcriptional capping (2). As chemical synthesis technology has advanced, the structure of cap analogs has evolved from first-generation standard cap structure analogs (m^7^GpppG, mCap) to second-generation ARCAs and third-generation Cap 1 analogs (CleanCap^®^). mCap has two free 3’-OH groups, allowing it to bind to the mRNA sequence in two orientations. Among these, the reverse-added cap structure has weak binding to eIF4E, preventing effective mRNA translation and resulting in low target protein production (59, 60). ARCA, based on the first-generation cap analogs, undergoes methylation modification to avoid reverse capping. Specifically, the third position 3’OH of ARCA is replaced by 3’-deoxy or 3’-O-methyl, leaving only one 3’-OH, which forces ARCA to bind to the mRNA sequence in a forward manner. The mRNA capped with ARCA exhibits higher translation efficiency than mCap (43, 61). During the co-transcriptional process, ARCA competitively binds to the mRNA sequence with GTP, reducing ARCA capping efficiency to approximately 60%-80% (14). ARCA co-transcriptionally generates a Cap 0 structure, which requires further methylation to form a Cap 1 structure (2). ARCA capping has limitations such as relatively low capping efficiency (60%-80%), Cap 0 structure formation after capping, a cap containing a non-natural O’-methyl at position C3, which can be recognized as a foreign sequence, and a requirement for mRNA transcripts to start with guanine (G). While the enzyme-based capping method can achieve a capping efficiency of 100% and yields a naturally unmodified cap structure, the process is costly and batch-to-batch variations exist. CleanCap^®^ Cap 1 analogs overcome these challenges associated with ARCA. CleanCap^®^ directly generates mRNA with a Cap 1 structure during transcription, achieving a capping efficiency of nearly 90-99%, which is higher than ARCA (14). The common CleanCap^®^ cap analogs are CleanCap^®^ Reagent AG (m7GpppA2’OMepG), CleanCap^®^ Reagent AG 3’OMe (m7G3’OMepppA2’OMepG), CleanCap^®^ Reagent AU (m7GpppA2’OMepU), and CleanCap^®^ M6 (m7G3’OMepppm6A2’OMepG) (2, 62). CleanCap^®^ Reagent AG, CleanCap^®^ Reagent AG 3’OMe, and CleanCap^®^ M6 are commonly used for nrmRNA vaccines. The application of these reagents requires the DNA template must start with an AG sequence immediately following the T7 promoter sequence (63–65). Compared to CleanCap^®^ Reagent AG, CleanCap^®^ Reagent AG 3’OMe not only results in higher expression levels *in vivo* but also achieves broader distribution and longer duration (64). CleanCap^®^ Reagent AU is a capping analog specifically designed for samRNA vaccines, requiring the DNA template’s 5’ initiation sequence to begin with AU (66). CleanCap^®^ M6 significantly enhances protein expression *in vivo* after mRNA capping, primarily due to its ability to inhibit the decapping process mediated by Dcp2 (mRNA decapping enzyme). Consequently, CleanCap^®^ M6 holds promise for improving the therapeutic efficacy of mRNA drugs while reducing the administered dosage (62). The BNT162b2 vaccine uses the CleanCap^®^ co-transcriptional capping technique (67). The co-transcriptional capping method is a single-step process with a high capping rate, requires only one purification step, simplifies mRNA production, and allows for easy scale-up in the manufacturing process.

#### 4.2.2 UTRs selection

UTRs include 5’ UTR and 3’ UTR, which do not encode proteins but mainly regulate mRNA stability, translation efficiency, nuclear export, and cellular localization, thereby directly affecting protein expression (68). The 5’ UTR is located upstream of the mRNA’s ORF, extending from the methylated guanine nucleotide cap at the mRNA start point to the AUG start codon. The 5’ UTR recruits ribosomes and participates in the small subunit scanning of the start codon, which regulates the translation of downstream ORF sequences (69–71). When designing 5’ UTRs, it is important to note the following: First, avoid the presence of start codon (AUG), and non-canonical start codons (CUG) in the 5’ UTR, as these codons may disrupt the normal translation process of ORF. Second, avoid the presence of highly stable secondary structures, as these can inhibit ribosome recruitment and codon recognition. Third, a shorter 5’ UTR is more conducive to mRNA translation. Lastly, bioinformatics tools should be used to predict mRNA translation efficiency based on the 5’ UTR sequence (14). In mRNA vaccine development, there are three strategies for optimizing the 5’ UTR. The first method is to simply extract the 5’ UTR of highly expressed human genes, such as from the human α-globin gene. The second is to use the natural 5’ UTR of the pathogen’s mRNA. These two strategies are based on the 5’ UTR optimized by natural selection, which can function in muscle cells. The third method is through the Systematic evolution of ligands by exponential enrichment (SELEX), which has been used to optimize the 3’ UTR but can also be used for 5’ UTR. For vaccines against epidemic infectious diseases, rapid development is paramount, so the first two methods seem more reasonable (69). The design of the 5’ UTR of BNT162b2 incorporates the 5’ UTR of human α-globin A1 and A2 and includes minor modifications to the shared Kozak sequence (72). The 5’ UTR of mRNA-1273 is V1-UTR, which is designed by Moderna (73). The 3’ UTR extends from the ORF termination codon to the front end of Poly (A) and regulates mRNA stability through enriched AU and GU elements. The 3’ UTR can also regulate the translation efficiency and subcellular localization of mRNA (74–76). The natural 3’ UTR of highly expressed genes is the preferred source for synthetic mRNA, such as the α and β subunits of hemoglobin, albumin, and heat shock protein 70 (2). The number of 3’ UTRs has an impact on mRNA. Studies have demonstrated that adding two 3’ UTR sequences in a series can improve mRNA stability and translation efficiency (77–79). The 3’ UTR of mRNA-1273 is the 3’ UTR of human α-globin A1, while the 3’ UTR of BNT162b2 is a combination of the 3’ UTR of the human AES/TLE5 gene, selected by SELEX, and the human mitochondrial 12S rRNA gene (MTRNR1), with two mutation sites introduced on AES/TLE5 (77).

#### 4.2.3 ORF optimization

An ORF is a segment of a nucleotide sequence in mRNA that commences with the initiation codon (AUG) and concludes with the termination codon (UAA, UAG, or UGA) and functions as the coding region for protein synthesis. The selection of codons within this region directly affects mRNA stability and translation efficiency (80, 81). Exogenous mRNA can often contain codons that the host cell infrequently employs, leading to decreased protein expression levels. Therefore, it is necessary to implement codon optimization, replacing these rare codons with synonymous ones more frequently used by the host cell (81). This optimization increases the translation efficiency of the ORF and reduces the likelihood of recognition by PRRs, thus minimizing innate immune responses (82). A study discovered that genes with a high GC content in mammalian cells display significantly superior expression efficiency-ranging from several times to a hundred times higher-compared to those with a low GC content. This is attributed to the more effective transcription of GC-rich genes, leading to the production of a greater number of stable mRNAs (83). Adjusting the GC content of the ORF can prevent the formation of secondary structures in mRNA, enhancing its stability and increasing *in vivo* protein expression levels (84). The structuring of mRNA plays a vital role in mRNA translation, with highly stable secondary structures and hairpin loops avoided as they can impede the entry, scanning, and elongation of ribosomes and may be recognized as PAMPs by the innate immune system. Increasing the GC content of mRNA vaccines can lower the amount of uridine due to the recognition of uridine-rich regions by RIG-I, thus activating the innate immune system to suppress protein expression (55). However, the expression of some proteins necessitates the presence of rare codons, slowing the progression of ribosomes to allow for proper protein folding (80, 85). Thus, different codon optimization strategies can be employed depending on the antigen. If mRNA vaccines, typically delivered via intramuscular injection, are codon-optimized preferentially for skeletal muscle, a more effective immune response can be achieved (2). The Kozak sequence is a specific nucleotide sequence surrounding the start codon of eukaryotic mRNA, which facilitates proper ribosome recognition and binding to the mRNA, thereby initiating translation (86, 87). Therefore, inserting a Kozak sequence after the 5’ UTR can enhance translation efficiency (88, 89).

#### 4.2.4 Poly(A) tails selection

**Figure 4:**
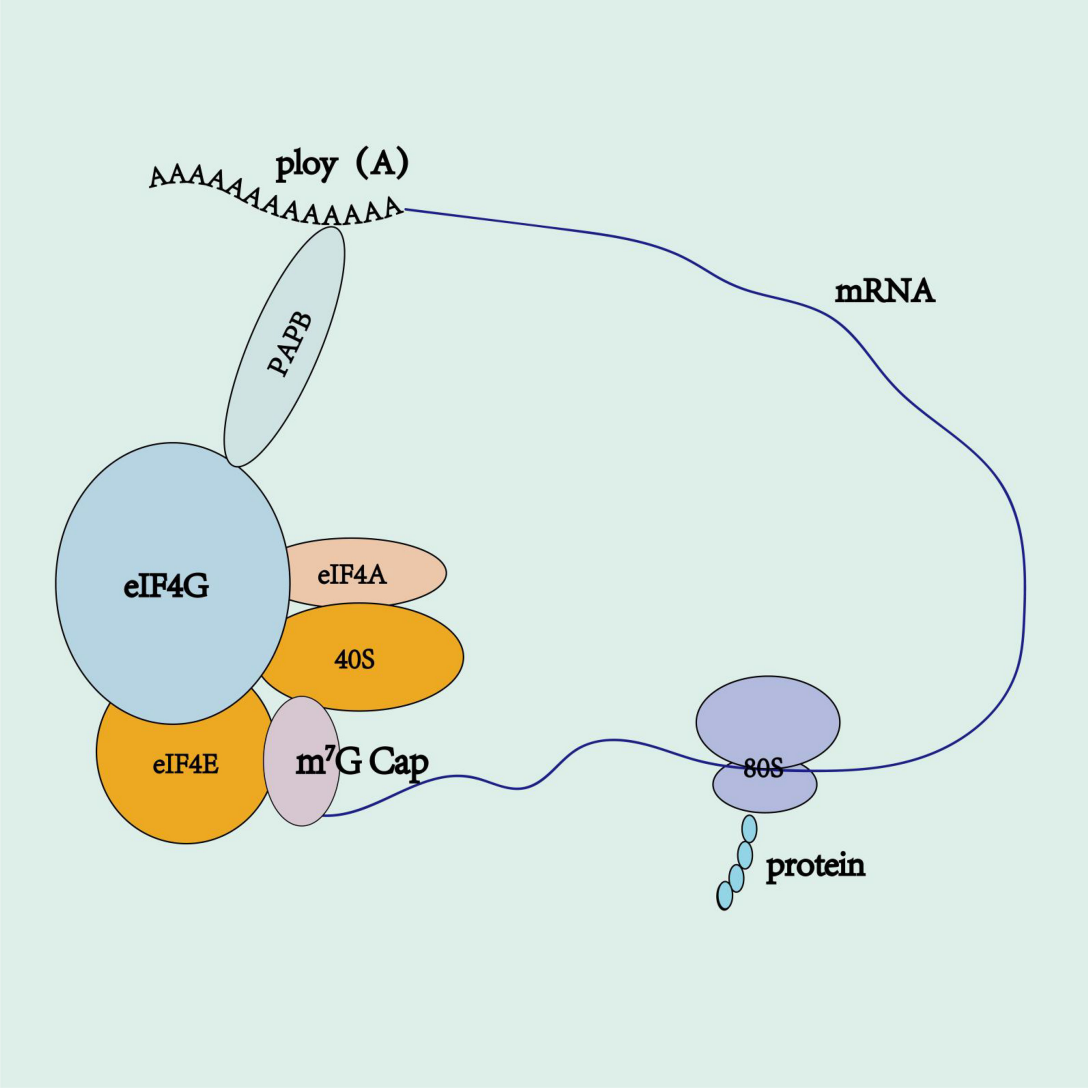
mRNA circular translation complex. 5’ cap binds to eIF4E, which, in turn, associates with eIF4G and eIF4A to form the cap-binding initiation complex eIF4F. The eIF4F complex recruits the 40S ribosomal small subunit, while the poly(A)-binding protein (PABP) interacts with eIF4G. Acting as an anchor, eIF4G directly links the 5’ cap and the poly(A) tail of mRNA, forming a “ closed-loop “ structure.

In eukaryotes, virtually all mRNAs have poly(A) tails (90). These tails safeguard mRNA from degradation, bolster mRNA stability, and augment translation efficiency (91). Eukaryotic mRNA features a circular translation complex and a 5′Cap that binds to eIF4E, which in turn forms the cap-binding initiation complex eIF4F with eIF4G and eIF4A (92–94). The eIF4F combines with the 40S ribosomal small subunit, while the cytoplasmic polyadenylate-binding protein (PABPC1) RRM1-2 links to eIF4G, using it as an anchor, to directly connect the cap and tail of mRNA. This forms a “ closed-loop “ structure, effectively deterring the “ decapping “ and “ de-tailing “ of mRNA, thereby amplifying mRNA stability, initiating translation, and favoring ribosome recycling (**Figure 4**) (93, 94). There is no straightforward linear relation between poly(A) tail length and the efficiency and stability of mRNA translation (91). An optimal poly(A) tail length can enhance these parameters, whereas a short tail will fail to stimulate mRNA translation (95, 96). Studies suggest that as the poly(A) tail length extends to 120 bp, the corresponding protein expression levels also rise, but there is no further enhancement beyond this length (2). Tailing of IVT mRNA utilizes two methods: a. enzymatic synthesis, which involves IVT performance followed by enzyme-catalyzed polyadenylation to append a length-uncontrolled poly(A) tail at the 3′ end of the mRNA; b. co-transcriptional synthesis, which designs a fixed-length poly(A) sequence on the DNA template and synthesizes an mRNA with a fixed-length poly(A) tail through IVT (2, 72, 78). Enzymatic tailing is influenced by reaction conditions such as temperature and enzyme quality, leading to uncertainty in the poly(A) tail’s length. Co-transcriptional tailing, the preferred method, maintains product homogeneity and accurate poly(A) tail length, reducing processing steps and cutting costs. The BNT162b2 vaccine applies a segmented poly(A) tail co-transcriptional method, connecting two tail structures A30 and A70 with a segment of ten nucleotides GCATATGACT. This method extends mRNA half-life and enhances translation efficiency more effectively than the long-chain poly(A) tails method (2). Studies show that N6-methyladenosine (m6A) modification of poly(A) tails stabilizes the variant surface glycoprotein transcript, further enhancing mRNA stability (97). The poly(A) tail typically consists of an adenine chain, but recent research indicates that mRNA tails with cytosine (C) can raise the level and persistence of mRNA expression. Additionally, cytosine (C) substitution can strengthen resistance to mRNA degradation, thereby prolonging its half-life (98).

#### 4.2.5 Nucleotide modification

IVT-synthesized ssmRNA is unstable, and some ssmRNA forms local dsRNA structures (99). When introduced to the body, dsRNA binds to the TLR3 receptors as ligands, while ssRNA binds to TLR7 and TLR8 receptors in a similar fashion, which activates the innate immune system (100, 101). Natural nucleotide modifications in human mRNA prevent it from being recognized by the immune system (102). However, as IVT mRNA lacks these chemical modifications, it can be recognized and degraded by the host immune system, calling for the chemical modification of its nucleotides (103).

Kariko et al. (104) employed pseudouridine to replace uridine for mRNA modification, resulting in an altered mRNA secondary structure. This alteration helps prevent recognition by the innate immune system and RNase degradation, thereby enhancing mRNA translation efficiency. Common nucleotide modification methods include pseudouridine (ψ), N1-methylpseudouridine (m1ψ), 5-methoxyuridine (mo5U), 2-thiouridine (s2U), 5-methylcytidine (m5C), and N6-methyladenosine (m6A) (2). Research has displayed that replacing original nucleotides with m6A and s2U can suppress TLR3 activation. Meanwhile, the substitution of original nucleotides with m5C, 5-methyluridine (m5U), s2U, m6A, and ψ can prevent TLR7 and TLR8 activation, assisting in avoiding activation of the innate immune system and promoting protein translation efficiency (2, 105).

Both BNT162b and mRNA-1273 vaccines utilize m1ψ to replace uridine for their modification (106). Kormann et al. (107) substituted 25% of mRNA cytidine with m5C and 25% of mRNA uridine with s2U. This modification promoted mRNA stability and heightened protein translation efficiency in mice. Therefore, swapping natural nucleotides with modified ones in the proper proportions can bolster mRNA stability and translation efficiency, but this technique also presents challenges. At present, mRNA vaccines often substitute natural nucleotides with chemically modified ones to maximize safety and stability, with m1ψ frequently used to replace uridine (2).

Epigenetic modifications of post-transcriptional mRNA also serve as a strategy to enhance translation and avoid innate immune responses. For instance, Arango et al. (108) reported that post-transcriptional modification with N4-acetylcytidine (ac 4C) in both *in vitro* and *in vivo* settings boosted mRNA translation.

## 5 Delivery vector development

### 5.1 Lipid nanoparticles

The mRNA molecule has a large molecular weight (approximately 10^4^-10^6^ Da), is hydrophilic, carries a negative charge, and is easily degraded by nucleases, making it difficult for it to cross the phospholipid bilayer of the cell membrane (109). Therefore, various delivery vectors for mRNA, such as lipid nanoparticles (LNPs), lipoplexes (LPX), Proteolipid vehicle have been developed. These vectors may deliver mRNA into human cells using different delivery methods. Once inside the cells, the mRNA is translated into the corresponding target protein, thereby activating cellular and humoral immunity (2). The function of the delivery system for mRNA vaccines includes: a. protection of the mRNA to reach the target site; b. assistance for the mRNA to enter the cells effectively; c. and release of the mRNA into the cytoplasm before reaching the lysosome. The delivery vectors of BNT162b2 and mRNA-1273 are both LNPs, which is currently considered the most advanced mRNA vaccine delivery system (110–112). LNP is a lipid nanovesicle with a lipid structure similar to that of a cell membrane. This tiny lipid droplet can deliver encapsulated nucleic acids and small molecule drugs into cells (19). LNP possesses many advantages, such as a simple formulation, modularity, biocompatibility, and high mRNA loading capacity (113). LNP consists of four sections: ionizable cationic lipid, PEGylated lipid, phospholipid, and cholesterol (8). The LNP preparation relies on the ability of self-assembly, that is, four components spontaneously form nanostructured entities via intermolecular interactions (19).

In LNPs, ionizable cationic lipids constitute 30-50% of total lipids, with commonly used examples including 1,2-Dioleoyl-3-dimethylammonium-propane (DODAP), 1,2-Dioleyloxy-3-dimethylaminopropane (DODMA), and DLin-MC3-DMA (113). These lipids pKa values are generally in the range of 6.0 to 7.0, allowing them to become positively charged under acidic conditions, bind to negatively charged mRNA, then encapsulating the mRNA within LNPs. Then, through hydrophobic interactions and van der Waals forces with other lipid components, they are assembled to form mRNA-LNP (19). After that, through buffer exchange, the mRNA-LNP solution is adjusted to neutral pH. During this process, ionizable cationic lipids become neutral, reducing aggregation caused by interaction with serum proteins. This decreases the likelihood of being phagocytosed and cleared by mononuclear macrophages, thus extending the drug’s half-life and attenuating the toxic side effects associated with traditional permanent cationic lipids (19, 34, 114). After being taken up by cells, mRNA-LNP become protonated in the acidic environment of endosomes (pH below the apparent pKa), leading to fusion between the LNP and the endosomal membrane, which results in membrane disruption and subsequent mRNA escape into the cytoplasm (115, 116). Studies have shown that LNPs with pKa values between 6.2 and 6.5 are beneficial for liver delivery of siRNA *in vivo*, while LNPs with pKa values between 6.6 and 6.9 are advantageous for intramuscular mRNA vaccines delivery (117).

PEG lipids constitute about 1.5% of the total lipids (34). Since LNPs are recognized as foreign by the body, they are easily cleared by monocytes and macrophages. PEG chains extend from the surface of LNPs, forming a hydrated layer that spatially shields the surface, preventing LNP aggregation and fusion, reducing serum protein adsorption, opsonization, and phagocytosis, thus extending the circulation half-life. Additionally, PEG promotes lipid connectivity. Although PEG can reduce serum protein adsorption and improve stability, it also diminish interactions with the cell membrane, leading to decreased cellular endocytosis. Furthermore, after endocytosis, PEG can reduce interactions between LNPs and endosomal membranes, affecting the release of mRNA into the cytoplasm. Therefore, precise control of its dosage and molecular weight is necessary (19, 34, 117–119).

Phospholipids can spontaneously organize into lipid bilayers and have a relatively high phase transition temperature. Therefore, they are used as structural lipids and can promote the fusion of LNPs with endosomal membranes, which is helpful for the release of mRNA. Phospholipids account for about 10-20% of the total lipids in LNPs. 1,2-Dioleoyl-sn-glycero-3-phosphoethanolamine (DOPE) can improve the transfection efficiency of mRNA and also affect the biodistribution of LNP *in vivo* (34, 120).

Cholesterol is also a structural lipid that exists in the shell of LNP and accounts for about 20 - 50% of the total lipids in LNPs. Cholesterol enhances the stability of particles by regulating membrane integrity and rigidity. The molecular geometry of cholesterol derivatives can further influence the delivery efficiency and biodistribution of LNPs (19, 34, 117, 118).

The particle size of LNPs affects their internalization, biodistribution, immunogenicity, degradation, and clearance. Controlling the particle size of LNPs also enables targeted delivery to specific tissues and cells. Typically, the optimal particle size range for LNPs is 20-200 nanometers, which provides ideal permeability and retention capabilities, allowing them to pass through stromal tissues. The components of LNPs also have certain drawbacks that require improvement. PEG is a significant potential source of peroxides and is generally unstable. Its degradation can lead to chain reactions and catalyze the degradation of ionizable lipids. Lipid degradation products can also react with mRNA, resulting in the formation of covalent mRNA-lipid adducts, which can affect mRNA bioactivity (34). Additionally, PEG-containing LNPs can bind to immunoglobulins on marginal zone B cells in the spleen, stimulating the production of anti-PEG IgM. Upon a second administration, the previously generated anti-PEG IgM binds to PEG on the LNPs, subsequently activating the complement system and enhancing the phagocytic activity of Kupffer cells, leading to increased clearance of the LNPs (121). Nogueira et al. (122) replaced traditional PEGylated lipids with polyaspartic acid lipids to create polyaspartic acid-functionalized LNPs, which demonstrated lower immunogenicity compared to conventional PEGylated LNPs, to avoid the adverse effects of PEG. Furthermore, phospholipid 1,2-distearoyl-sn-glycero-3-phosphocholine (DSPC) and ionizable lipids are highly susceptible to temperature- and pH-dependent hydrolysis during storage (34).

### 5.2 LPX

LPX is primarily composed of cationic lipids like N-[1-(2,3-dioleoyloxy)propyl]-N,N,N-trimethylammonium (DOTMA), 1,2-dioleoyl-3-trimethylammonium propane (DOTAP), and dimethyldioctadecylammonium bromide (DDAB) and cholesterol. Cholesterol stabilizes the liposome structure (123). Due to their amphiphilic nature, cationic lipids self-assemble into vesicular structures, with mRNA encapsulated within or between lipid layers. The properties of LPX, including size, charge, and stability, can be adjusted by modifying the lipid types, component ratios, and the cationic lipid-to-anionic mRNA ratio (124). Kranz et al. (125) showed that adjusting the ratio of cationic lipids to mRNA affects the charge ratio of the mRNA-LPX complex. When the charge ratio is 1.3: 2, the complex becomes negatively charged, exhibiting good stability, resistance to degradation, and spleen-targeting specificity. Clathrin-mediated endocytosis and caveolae-mediated endocytosis play critical roles in the cellular uptake of LPX. The mechanisms of lipid flip-flop and multiple transient pore formation are considered crucial for LPX-mediated endosomal escape (124). BioNTech’s BNT111 cancer vaccine, which uses LPX as a carrier to encode tumor-associated antigens and elicit immune responses against tumor cells (126).

### 5.3 Proteolipid vehicle

LNP delivers mRNA into the cytoplasm through endosomal escape, a process that can be cumbersome and has certain limitations. In addition, LNP struggles to encapsulate larger molecules like DNA, hence its use is restricted to RNA-based gene therapy methods. To navigate these drawbacks, Brown et al. (127) conceived a proteolipid vehicle (PLV). The PLV employs scalable microfluidic mixing technology to integrate fusion-associated small transmembrane (FAST) proteins derived from fusogenic avian reovirus into a well-tolerated lipid formulation. The FAST protein family includes p10, p13, p14, p15, p16, and p22. After screening the FAST protein library, p14endo15 was identified and incorporated into the 41N lipid formulation, resulting in the development of FAST-PLV. This chimera consists of the extracellular domain of p14, a single transmembrane domain (TMD), and the intracellular domain of p15. This structural composite enhances its fusogenic interaction with cell membranes. By fusing PLV with cell membranes, mRNA or pDNA can be delivered to the cytoplasm. Adeno-associated virus (AAV) vectors have demonstrated promising results, but their small payload capacity and the immune response to AAV restrict their use. In contrast, FAST-PLV has a large payload capacity. The introduction of FAST proteins into lipid formulations boosts the expression of mRNA and pDNA, and their low immunogenicity allows for multiple administration. Additionally, PLV displays a robust capability for extrahepatic delivery, making it potentially significant for clinical use in treating advanced cancers.

### 5.4 Targeting and delivery efficiency of delivery vectors

An ideal delivery vector should possess targeting and high efficiency to allow mRNA to function efficiently in specific cells and organs, thus avoiding the side effects of systemic exposure. Traditional LNPs have liver-targeting properties. When these enter the bloodstream, apolipoprotein E (ApoE) in the serum adsorbs onto the surface of the LNPs, which then bind to LDL receptors on the surface of liver cells, mediating LNPs into liver cells (128). On this basis, optimizing the various components of lipid nanoparticles (LNPs) can improve the efficiency of LNPs and enable targeted delivery to non-liver tissues.

Optimizing ionizable lipids: The lipid consists of three key components: an amine head group, a hydrophobic tail, and a linker. The chemical diversity within each of these components enables the creation of various distinct lipid structures through combinatorial chemistry. High-throughput screening (HTS) is typically performed both *in vitro* and *in vivo* to identify the top-performing ionizable cationic lipids from hundreds of candidates (117). Zhou et al. (129) identified 5A2-SC8 LNP from over 1,500 modular degradable candidates through HTS. This LNP efficiently delivered let-7g miRNA *in vivo*, significantly inhibiting liver cancer tumor growth and extending survival. Beyond HTS, exploring the structure-activity relationship (SAR) of ionizable cationic lipids or conjugating targeting moieties can further enhance targeted delivery. Qiu et al. (130) discovered that O-series LNPs (with ester bonds in their tails) tend to deliver mRNA to the liver, while N-series LNPs (with amide bonds in their tails) selectively deliver mRNA to mouse lungs. By adjusting the head structure, different lung cell types can be targeted. Zhao et al. (131) constructed and screened lipids containing imidazole or imidazole-like groups, analyzing their SAR. The best lipid screened can deliver mRNA into primary T lymphocytes, achieving a gene recombination rate of 8.2%.

Optimizing phospholipids: Studies have found that phospholipids can alter organ tropism. Zwitterionic phospholipids primarily achieve liver-targeted delivery, while anionic phospholipids promote spleen-targeted delivery (117). Phosphatidylserine (PS) is a signal released by senescent cells to mononuclear macrophages to promote phagocytosis of senescent cells. Luozhong et al. (132) utilized dioleoylphosphatidylserine (DOPS), a lipid containing the PS structure, as a helper lipid to construct LNPs. PS enhances the endocytosis of LNPs by monocytes, facilitating targeted delivery to secondary LNs; Optimizing cholesterol: Studies have found that 20α-hydroxycholesterol (20α-OH) LNPs have a fivefold greater capacity to deliver mRNA to endothelial cells and Kupffer cells compared to hepatocytes (117).

Optimizing PEG lipids: While PEG can prolong the circulation time of LNPs, it also reduces their contact with cells, leading to decreased transfection efficiency. Therefore, precise control of PEG’s molecular weight and its proportion within LNP components is essential. PEG lipids with a molecular weight of 2,000 Da at a molar ratio of 1.5% in LNPs point to minimizing the impact of PEG on mRNA delivery efficiency, while also significantly enhancing the stability and circulation time of the LNPs (133).

Cheng et al. (134) reported a selective organ targeting (SORT) method, which involves adding a fifth component to achieve tissue-specific mRNA delivery. The specific mechanism of SORT is as follows: a) PEG desorbs from the LNP surface, leading to the exposure of potential SORT molecules; b) different serum proteins recognize SORT molecules and adsorb onto the LNP surface; c) the surface-adsorbed proteins bind to homologous receptors highly expressed on specific tissue cells, mediating the entry of LNP-mRNA into target cells. Experiments have shown that the added SORT molecules, which are ionizable cationic lipids (e.g., DODAP), can target the liver by adsorbing ApoE, as the LDL-R receptor, which is highly expressed on liver cell surfaces, binds to ApoE. Adding SORT molecules like anionic lipids (e.g., 18PA) can target the spleen by adsorbing β2-glycoprotein I (β2-GPI), which binds to phosphatidylserine (PS), an anionic lipid exposed by aging red blood cells. The spleen’s function involves the degradation of aging red blood cells. Adding SORT molecules with permanently cationic lipids containing quaternary ammonium heads (e.g., 1,2-dioleoyl-3-trimethylammonium propane) can target the lungs by adsorbing vitronectin (Vtn), as the αvβ3 integrin receptor for Vtn is highly expressed on pulmonary endothelial cells (135).

Nucleoside-modified mRNA effectively avoids immune recognition and uncontrolled inflammation. While these modifications enhance tolerance and translation efficiency, they significantly impair innate immune responses and weaken the activation of dendritic cells, which are the primary receptor cells for mRNA-LNP vaccines, thus diminishing adaptive immunity (136). Researchers have discovered that certain LNPs possess intrinsic adjuvant activity, prompting modifications based on this property. Han et al. (136) chemically synthesized the adjuvant liposome C12-TLRa, based on TLR 7/8 agonists, and partially replaced the ionizable lipids in C12-113 LNPs, introducing a fifth component to the LNPs. This component induces innate immune activation via TLR7/8, promoting the maturation of APCs and effectively stimulating strong neutralizing antibodies, robust Th1-skewed cellular immunity, and significant B cell and long-lived plasma cell responses, while exhibiting good tolerability in mice. Additionally, LNPs incorporating this component interact with TLR7/8 receptors on endosomal membranes, effectively facilitating the release of mRNA into the cytoplasm.

Targeted delivery vehicles can also conjugate antibodies, ligands or activated proteins to LNPs to enhance their targeted delivery capability. Parhiz et al. (137) conjugated platelet endothelial cell adhesion molecule-1 (PECAM-1) antibodies with mRNA-LNPs to prepare lung-targeted LNPs. Compared to non-targeted LNPs, lung-targeted LNPs exhibited approximately a 200-fold increase in mRNA delivery efficiency to the lungs, resulting in a 25-fold increase in protein expression.

## 6 Factors of mRNA vaccine stability

The stability of mRNA vaccines is associated with multiple factors such as mRNA length and structure, excipients, the LNP delivery system, and the manufacturing process. There is a negative correlation between mRNA length and half-life. Guillaume et al. (138) reported that the second-generation Moderna COVID-19 mRNA vaccine, mRNA-1283, encoding the shorter N-terminal and receptor-binding domain of the severe acute respiratory SARS-CoV-2 spike protein, has significantly improved stability compared to the first-generation mRNA-1273 vaccine that encodes the full-length spike protein. As a result, the shelf life of mRNA-1283 at 2°C-8°C is extended from 6 months to 12 months. Additionally, modifying and optimizing the structure of mRNA components can enhance stability. Sucrose is a commonly used excipient that reduces the crystallization temperature and ice crystal formation in aqueous solutions. In the formulations of Moderna’s and Pfizer/BioNTech’s COVID-19 mRNA vaccines, sucrose is also employed as a stabilizer and cryoprotectant (34). Further optimization of the LNP delivery system should be pursued to address and improve the previously mentioned instability factors. The production process for mRNA-LNPs primarily involves preparation techniques, pH conditions, buffer systems, and lyophilization techniques. The specific process includes dissolving mRNA in a low-pH aqueous buffer (approximately pH 4.0) and mixing it with an ethanol solution of hydrophobic lipids through microfluidics to form stable mRNA-LNPs with a low polydispersity index. Initially, these mRNA-LNPs contain 25%-50% ethanol at a low pH. Therefore, the product undergoes further dialysis and buffer exchange using tangential flow filtration, followed by sterilization through a 0.2-µm sterile filter before being placed into sterile containers. The final product can be either lyophilized or directly filled (113). mRNA-LNPs are principally prepared using microfluidics or microjet techniques, with the resulting product containing about 1% ethanol. This ethanol can induce lipid membrane fusion, leading to mRNA leakage and affecting vaccine stability (34). mRNA is more stable in weakly alkaline environments. Bauer et al. (139) observed that the hydrolysis rate of nucleic acids significantly increases when the pH drops from 7.0 to 6.5. Consequently, the pH of the COVID-19 mRNA vaccines from Moderna and Pfizer/BioNTech is maintained between 7 and 8. Pfizer/BioNTech’s COVID-19 mRNA vaccine uses Tris-HCl to stabilize nucleic acids and neutralize hydroxyl radicals. Structural analysis of mRNA-LNPs shows that mRNA, ionizable cationic lipids, and water are located in the core of the LNP, while other neutral excipient lipids mainly reside on the outer shell. Lyophilization improves the stability of mRNA-LNP formulations by reducing the product’s moisture content. During the lyophilization process, the product structure is under stress. Cryoprotectants are commonly added to protect the product from freezing or drying stress and enhance its stability during storage. Commonly reported cryoprotectants for lyophilized nanoparticles include trehalose, sucrose, glucose, and mannitol. A lyophilized cytomegalovirus mRNA vaccine (mRNA-1647) developed by Moderna is currently in Phase 3 clinical trials and is reported to have a shelf life of up to 18 months at 5°C (34).

## 7 Biomedical application

### 7.1 Application of mRNA vaccine in infectious diseases

The challenge of vaccine production lies in producing sufficient doses in a short period for infectious diseases, an issue that mRNA vaccines can address. Since 1980, nearly 90 human-infecting pathogens have been discovered worldwide. Reports now suggest the emergence of two new human-infecting viruses annually, emphasizing the urgent need for vaccines to prevent infectious diseases (140, 141). Intracellular parasitic bacteria like *Brucella* elude immune responses due to their capacity to survive and multiply within cells. Antibiotics often show limited effectiveness against intracellular bacteria and the resistance of these bacteria to antibiotics is growing annually. mRNA vaccines, which can penetrate cells, offer the potential to activate intracellular immune responses or generate substances similar to antibiotics within cells, potentially eliminating intracellular parasitic bacteria like *Brucella*. mRNA vaccines can also be designed to target multiple antigens and incorporate pro-inflammatory cytokines to enhance their efficacy and improve resistance to emerging variants and co-infections (113, 142). Currently, clinical trials of mRNA vaccines are making significant progress.

#### 7.1.1 mRNA vaccine in viral diseases

In 1993, Martinon et al. (143) first demonstrated that liposome-encapsulated mRNA encoding the influenza virus nucleoprotein (NP), could induce virus-specific cellular immune response *in vivo*, offering a new direction for vaccine development. In 1994, Zhou et al. (144) constructed a recombinant RNA vaccine resembling a saRNA vaccine by utilizing modified Semliki Forest virus (SFV) replicons to express the influenza NP. This vaccine exhibited strong immunogenicity and effectively stimulated both cellular and humoral immune responses in mice. Subsequently, research into mRNA as immunotherapies, particularly in oncology, expanded with *in vitro* and *in vivo* assays employing both protected and unprotected (“naked”) mRNA (145). Despite many mRNA vaccines progressing through preclinical and clinical stages over an extended period, none reached practical application. This changed in December 2019 with the outbreak of COVID-19, which significantly accelerated the development process of mRNA vaccines. Pfizer/BioNTech’s BNT162b2 and Moderna’s mRNA-1273 received approval and were rapidly deployed to combat the pandemic.

##### 7.1.1.1 COVID-19 mRNA vaccine

In the Phase III clinical trial of the BNT162b2 vaccine, participants received two doses, each of 30 μg, administered 21 days apart. The results indicated that the vaccine’s efficacy in preventing SARS-CoV-2 was as high as 95% 28 days (111). after the first dose. The FDA granted emergency use authorization for the BNT162b2 vaccine on December 11, 2020, making it the first officially approved mRNA vaccine. In the Phase III clinical trial of mRNA-1273, participants received two doses, each 100 μg, administered 28 days apart. After the first injection, the vaccine demonstrated an efficacy of 94.1% after 42 days and was deemed highly safe (146). On December 18, 2020, the FDA approved emergency use authorization for the mRNA-1273 vaccine-the second approved mRNA vaccine. The design of vaccines relies heavily on the target antigens selected. The trimeric spike (S) protein on the surface of SARS-CoV-2, central to the virus’s host cell invasion, has been chosen as the primary antigen for vaccine design.

The S protein of wild-type SARS-CoV-2 is cleaved into the S1 and S2 subunits by the furin enzyme and transmembrane serine protease 2 (TMPRSS2) during the infection process. The S1 subunit’s receptor-binding domain interacts with the host cell’s angiotensin-converting enzyme 2 (ACE2) receptor, facilitating viral entry. The S2 subunit mediates the fusion between viral and host cell membranes. Currently, there are two common strategies exist in the design of the COVID-19 S protein: the 2P mutation (which substitutes two amino acids in the S2 subunit with proline to stabilize the prefusion conformation of the S protein) and alteration to the S1/S2 cleavage site mutation (which deletes or alters the cleavage site sequence to prevent S protein cleavage in the host cell, thereby maintaining its structural stability). The S protein can remain in its intact form for a longer period through these operations, continuously an extended duration, continually stimulating the immune system, and eliciting a robust immune response (2).

In Japan, ARCT-154, a saRNA vaccine, has been approved for COVID-19 immunization, marking the first non-emergency use authorization for an saRNA platform. The saRNA sequence encodes the SARS-CoV-2 spike (S) antigen and nsP1–4 derived from the Venezuelan equine encephalitis virus genome. Delivered to patient cells via LNPs, the translated S protein triggers an immune response, while nsP1–4 enable the self-replication of the saRNA, thereby increasing antigen expression levels and prolonging the duration of expression. Clinical trials have demonstrated that ARCT-154 exhibits safety comparable to traditional mRNA vaccines. Compared to the homologous mRNA vaccine, ARCT-154 elicits a stronger immune response against the Omicron BA.4/5 variant, characterized by higher neutralizing antibody titers that remain elevated six months post-booster and strong immunogenicity in individuals aged 65 and above. ARCT-154 is administered at a dose of 5 μg, merely one-sixth of the standard 30 μg dosage. Despite not utilizing modified nucleotides to reduce reactogenicity, ARCT-154 achieves high immunization efficiency through the self-replicating nature, the adjuvant effects of LNPs, and the unmodified RNA (147).

##### 7.1.1.2 Syncytial virus mRNA vaccine

Moderna’s mRNA-1345 vaccine against respiratory syncytial virus (RSV), designed for adults aged 60 and above, presented an efficacy rate of 83.7% in Phase 3 clinical trials. It effectively prevents RSV-caused lower respiratory tract infections (148). On May 31, 2024, the FDA approved the mRNA-1345 vaccine for the prevention of RSV infection – the first mRNA vaccine approval for a disease other than COVID-19.

mRNA-1345 encompasses a nucleotide-modified mRNA sequence encoding the membrane-anchor RSV F glycoprotein (RSV-A2 strain protein sequence). This protein is engineered to stabilize in the pre-fusion (preF) conformation and encapsulated in LNPs. The F protein, conserved across different strains and antigenic subtypes (RSV-A and RSV-B), is the chief antigenic target for the creation of protective neutralizing antibodies.

F protein has two predominant conformational states: pre-fusion (preF) and postfusion (postF). A transition from the unstable preF state into the relatively stable postF state facilitates viral fusion with the host cell membrane. As all known epitopes that can elicit neutralizing antibodies are represented in the preF conformation, it prompts higher neutralizing antibody responses in both animal models and humans compared to the postF conformation. Thus, stabilizing the F protein in the preF conformational state is essential in vaccine design (149).

##### 7.1.1.3 Influenza mRNA vaccine

Currently, seasonal influenza vaccines are primarily produced using eggs, cells, or recombinant protein manufacturing platforms. However, the overall effectiveness varies across different populations and seasons. When vaccines are well-matched to circulating strains, effectiveness in the general population can reach 40% to 60%. During the 2021-2022 season, the effectiveness against acute respiratory infections requiring medical attention caused by the predominant A/H3N2 strain was only 16%. The reduced effectiveness is influenced by various factors, including population age, low immunogenicity, and strain mismatch. Strain mismatch is mainly attributed to the lengthy production cycle and mutations arising when the virus propagates in eggs or cells used for vaccine production. mRNA-1010 vaccine, a quadrivalent seasonal influenza vaccine currently under clinical development. This LNP-mRNA encodes the hemagglutinin (HA) surface glycoproteins of four influenza strains recommended by the WHO: A/H1N1, A/H3N2, B/Victoria, and B/Yamagata. A Phase 1/2 randomized clinical trial evaluating the safety and immunogenicity of mRNA-1010 in healthy adults has shown promising initial results, demonstrating good safety and immunogenicity potential. Compared to traditional egg-or cell-based influenza vaccine production methods, mRNA-1010 offers the significant advantage of rapid adaptation to strain changes, effectively avoiding mismatches caused by mutations during virus propagation in eggs or cells. This innovative approach has the potential to overcome the limitations of traditional seasonal influenza vaccines, such as strain mismatch, providing a more effective solution for influenza prevention (150).

##### 7.1.1.4 Cytomegalovirus mRNA vaccine

Cytomegalovirus (CMV) is globally prevalent, with seroprevalence rates reaching up to 100% in Africa and Asia, and 80% in Europe and North America. CMV can cause multisystem damage and be transmitted through multiple routes, especially in newborns and immunocompromised individuals, and is a common cause of congenital infections. There is currently no approved CMV vaccine, and existing antiviral drugs do not improve neurodevelopmental outcomes in infants with congenital infections. The development of a CMV vaccine faces multiple challenges, such as the virus’s ability to remain latent in the body, its intercellular transmission, strain diversity, and the lack of suitable natural models. The mRNA-1647 vaccine contains six mRNA sequences encoding key CMV antigens, including glycoprotein B (gB) and the pentameric complex. This vaccine prompts human cells to produce antigens and trigger an immune response. It induces significant neutralizing antibody titers in fibroblasts and epithelial cells and activates T cells and memory B cells. Phase 1 clinical trials focused on evaluating the vaccine’s safety, reactogenicity, and immunogenicity. Participants received 3 doses of the vaccine, ranging from 30-300 µg, or a placebo. The results indicated that the vaccine is safe and induces both cellular and humoral immune responses. Phase 2 aims to further optimize the vaccine dose, assessing 50 µg, 100 µg, and 150 µg doses. The results showed that the 100 µg dose exhibited acceptable safety and good immunogenicity in both seronegative and seropositive participants. Phase 3 (CMVictory) is currently evaluating the efficacy, safety, and immunogenicity of the 100 µg dose in 7454 seronegative women aged 19-40. Immunogenicity and safety markers are being monitored, with completion expected in 2026. An additional extension study is underway to assess the long-term immune effects of the vaccine (151).

#### 7.1.2 mRNA vaccine in bacterial infectious diseases

The development of bacterial vaccines is typically more challenging than that of viral vaccines. First, there are more potential vaccine targets because bacterial genomes are generally larger than viral ones. Second, there are non-protein vaccine targets, such as sugars, to which mRNA vaccines are ineffective. Also, there is a higher degree of antigen variation. Moreover, the immune response expected from bacterial vaccines often differs from that induced by viral vaccines. For viruses, inducing neutralizing antibodies (nAbs) is usually sufficient to effectively prevent infection. However, extracellular bacteria, such as *Streptococcus pneumoniae*, *Escherichia coli*, and *Staphylococcus aureus*, can be targeted effectively through immune mechanisms like complement-mediated killing and antibody-mediated phagocytosis. Furthermore, while it might not be essential for most viral targets, inducing a robust cellular immune response is often critical for effectively targeting intracellular bacteria (36).

##### 7.1.2.1 Tuberculosis mRNA vaccine

The currently available tuberculosis vaccine, Bacillus Calmette-Guérin, has low efficacy and poor durability and often causes adverse events. Therefore, developing a safer and more effective tuberculosis vaccine remains an unfulfilled clinical need (36). In 2022, Larsen et al. (152) designed a self-amplifying mRNA vaccine encoding ID91 that stimulated the production of CD4^+^ Th1 cells in mice, significantly reducing bacterial load in the lungs. The Th1 subset of CD4⁺ T cells secrete cytokines such as IFN-γ, IL-2, and TNF-α. These cytokines play pivotal roles in various critical immune processes, including promoting cellular immunity, upregulating the expression of MHC molecules on dendritic cells to enhance antigen presentation efficiency, and facilitating the generation of M1-like macrophages to drive effective immune responses (153, 154). The gene encoding Mycobacterium tuberculosis-related antigens was cloned into the Venezuelan equine encephalitis virus replicon (repRNA) backbone, which contains a SGP. This was done to construct saRNA. Structural engineering was applied to bestow the vaccine with specific advantages. The fusion protein encoded by the ID91saRNA vaccine includes four M.tb antigens: Rv3619, Rv2389, Rv3478, and Rv1886, with the intent to induce immune responses against these antigens. The saRNA is fused with delivery vehicles such as nanostructured lipid carriers to provide stability and protect the RNA from degradation by ribonucleases while avoiding additional immune events triggered by the host’s reaction to exogenous vectors associated with viral delivery. Considering the presence of different strains in various global regions, the selection of vaccine antigens needs flexibility, allowing for adjustments based on regional necessities to enhance the vaccine’s efficacy in different areas. As such, the antigens in the saRNA can be substituted to better match the regional strains’ characteristics, thereby improving the protective efficacy of the vaccine.

##### 7.1.2.2 Brucellosis mRNA vaccine

Currently, there is no approved human vaccine for brucellosis. Existing animal vaccines are unsuitable for humans due to residual virulence, which poses potential risks (155). Zhu et al. (156) employed a reverse vaccinology strategy to design mRNA vaccines targeting *Brucella* and *Mycobacterium tuberculosis*. They selected the Omp25 and Omp31 proteins from *Brucella* and the MPT70 and MPT83 proteins from the H37Rv (L4 strain) of *M. tuberculosis.* They predicted helper T lymphocyte (HTL), cytotoxic T lymphocyte (CTL), linear B cell (LB), and conformational B cell (CB) epitopes by bioinformatics tools. These epitopes were comprehensively assessed for their allergenicity, toxicity, and antigenicity. Molecular docking analyses were also performed to assess interactions with MHC, enabling precise identification of optimal epitopes for vaccine construction. Subsequently, various properties of the constructed mRNA vaccine were analyzed, including physicochemical characteristics, secondary and tertiary structure, molecular docking, molecular dynamics simulations, mRNA secondary structure prediction, codon optimization, and in silico cloning. These evaluations systematically assessed the feasibility and efficacy of the vaccine. Similarly, Shi et al. (157) also applied reverse vaccinology to design a multi-epitope LptD-BTuB mRNA vaccine against *Brucella*. However, no *in vitro* or *in vivo* studies have yet been conducted to evaluate its immunogenicity.

##### 7.1.2.3 Lyme disease mRNA vaccine

Lyme disease, caused by *Borrelia burgdorferi*, is a zoonotic disease transmitted to humans primarily through tick bites and can lead to severe complications such as arthritis, myocarditis, and facial paralysis (158). Concerning lyme disease as a common tick-borne infection in the United States with no available human vaccine, Pine et al. (159) developed an mRNA-LNP platform-based vaccine using *B. burgdorferi*’s OspA as the candidate antigen. Compared to the aluminum-adjuvanted OspA protein subunit vaccine, a single immunization with the OspA mRNA-LNP vaccine in mice elicited stronger humoral and cellular immune responses, effectively protecting against bacterial infection.

##### 7.1.2.4 Diphtheria-tetanus-pertussis mRNA vaccine

Wolf et al. (160) developed an mRNA-DTP vaccine encoding multiple critical antigens, including pertussis-related PTX-S1, FHA3, FIMD2/3, PRN, as well as specific fragments of diphtheria toxin (DT) and tetanus toxin (TT). To enhance efficacy, some antigens were optimized and modified. In the mRNA-DTP-10 vaccine, which includes 10 antigens, additional antigens such as RTX, TCFA, SPHB1, and BRKA were encoded, with modifications to prevent glycosylation. These disease-related antigens, such as PTX-S1, FHA3, FIMD2/3, PRN, DT, and TT, enable the immune system to recognize features of various pathogens, thereby inducing specific antibodies and cellular immunity against different antigens. This broadens the immune defense against pertussis, diphtheria, and tetanus. Furthermore, the inclusion of RTX prompts the production of antibodies targeting adenylate cyclase toxin, neutralizing its toxic effects on immune cells. TCFA and SPHB1, associated with bacterial colonization and pathogenicity, induce antibodies that mitigate bacterial adhesion and virulence. Additionally, mRNA encoding BRKA elicits anti-BrkA antibodies, which block BrkA’s inhibitory effect on complement activation.

#### 7.1.3 mRNA vaccine in parasitic diseases

Pathogenic parasites, a diverse group of eukaryotic organisms, cause over one million deaths annually. However, the development of vaccines against these pathogens is challenging, primarily due to the complexity of eukaryotic cells as vaccine targets and their capacity to evade both innate and adaptive immune responses (36). While mRNA vaccines have demonstrated promise, they also come with significant challenges.

##### 7.1.3.1 Malaria parasite mRNA vaccine

In 2021, Mallory et al. (161) immunized mice with mRNA encoding the *Plasmodium falciparum* circumsporozoite protein (PfCSP) of the malaria parasite, using LNP encapsulation, and found that it inhibited the onset of malaria in these mice. PfCSP is a major immunodominant coat protein during the invasion stage of the malaria parasite, playing a crucial role in mediating host cell invasion, rendering it a chosen antigen for vaccine design. In addition to these attributes, the utilized mRNA was codon-optimized and included a signal peptide.

##### 7.1.3.2 *Toxoplasma gondii* mRNA vaccine

Luo et al. (162) developed a vaccine using a saRNA vector, pRREP, based on the SFV genome. They inserted the *T. gondii* nucleoside triphosphate hydrolase II (NTPase-II) gene into the vector, creating the saRNA vaccine RREP-NTPase-II, which was subsequently encapsulated in LNPs. The vaccine activates both humoral and cellular immune responses, providing protective effects against *T. gondii* infection.

##### 7.1.3.3 *Ixodes scapularis* tick mRNA vaccine

Sajid et al. (163) developed an mRNA vaccine (19ISP) encoding 19 types of salivary proteins from the *I. scapularis* tick. The 19ISP vaccine was injected multiple times to guinea pigs, which induced both humoral and cellular immune responses. In terms of humoral immunity, the guinea pig sera produced specific IgG antibodies against 10 salivary proteins, including Salp14 and Salp15. As for cellular immune, the vaccine activated various immune-related signaling pathways, such as TCR and BCR pathways, leading to the secretion of cytokines like IFN-γ and TNF-α from peripheral blood mononuclear cells. This vaccine effectively prevented the transmission of Lyme disease pathogens.

### 7.2 Application of mRNA vaccine in cancer

Conry et al. (164), in 1995, demonstrated that mRNA vaccines containing the carcinoembryonic antigen gene, when administered via muscular injection, trigger an anti-tumor immune response in mice. As mRNA vaccines and their delivery systems have matured, mRNA has emerged as a promising platform for cancer treatments. Cancer vaccines, however, require careful consideration of several key factors. For instance, the immune-suppressive tumor microenvironment can be transformed by expressing specific tumor suppressor proteins. Current mRNA delivery systems fail to reach all cancer cells in patients, requiring the optimization of these systems. There is, therefore, an increasing interest in using mRNA as a therapeutic vaccine to train the immune system to locate and destroy cancer cells. It is essential to select suitable antigens, given the significant antigen variability between individuals, for inducing a highly tumor-specific immune response. Even though an antigen can stimulate a cellular immune response, the suppressive tumor microenvironment may obstruct T cell infiltration into the tumor, potentially leading to T cell exhaustion. Hence, therapeutic vaccines may need to be used alongside other therapies to combat the suppressive microenvironment, like immune checkpoint inhibitors. mRNA cancer vaccines, when used independently, can be a reliable treatment method for early-stage cancers. However, the highly immunosuppressive tumor microenvironment in late-stage cancer patients can undercut the efficacy of mRNA monotherapy. Conversely, blending therapeutic cancer mRNA vaccines with other immunotherapies permits the introduction of mRNA-encoded immune therapy-related proteins, which include antibodies, cytokines, ligands, tumor suppressor proteins, and other functional proteins. Unlike preventive vaccines for infectious diseases, which primarily offer protection against infections through robust humoral immunity, therapeutic cancer vaccines have to ensure the induction of sturdy cellular immune responses to fully eradicate cancer cells. Even though preventive cancer vaccines exist, the FDA has approved only two of them so far, which target viruses known to cause cancer – human papillomavirus (HPV) and hepatitis B virus (142, 165).

The function of mRNA cancer vaccines is largely to elicit cellular immunity through tumor antigens. There are two kinds of tumor antigens: tumor-specific antigens (TSAs), found only on tumor cells, and tumor-associated antigens (TAAs), which both regular and tumor cells carry (113). For instance, in a clinical trial (NCT02410733), 119 melanoma patients received the BNT111 mRNA vaccine containing four TAAs: New York esophageal squamous cell carcinoma 1 (NY-ESO-1), melanoma antigen family A3 (MAGE-A3), transmembrane phosphatase with tensin homolog (TPTET), and tyrosinase. Despite mild flu-like side effects, patients who took monthly doses of the vaccine showed sustained and robust cellular immune responses to tumor antigens lasting over a year (125, 166). The FDA granted fast-track designation to the BNT111 vaccine based on these successful trials for melanoma treatment (166). It can be used concurrently with cemiplimab (167). The four TAAs in the BNT111 vaccine have optimized mRNA sequences for translation in immature dendritic cells. Each sequence includes a signal peptide, the tetanus toxic CD4^+^ epitopes (P2 and P16), as well as the MHC class I transport domain to augment human leukocyte antigen presentation and immunogenicity (165). However, relying on TAAs as tumor antigens is somewhat limited because they are also expressed in normal tissues. Immune tolerance mechanisms and the potential risk of off-target effects leading to autoimmune toxicity might hinder the development of an effective anti-tumor immune response (142). The mRNA-4157/V940 vaccine, encoding 34 *TSA* genes, managed to reduce the post-operative recurrence and mortality rate of cancer patients by 44% compared to pembrolizumab alone when combined with it. The FDA bestowed Breakthrough Therapy designation on it for adjuvant therapy in patients with wholly resected high-risk melanoma (168). Nevertheless, only a small portion of somatic mutations in cancer cells can be recognized by spontaneously generated T cells, and the effectiveness of these novel epitopes in mediating anti-tumor effects differs, posing challenges to their precise application (142). In response to the limited number of neoantigens and loss of antigen targets, Trivedi et al. (169) established an immunogenomics pipeline called the “Open Reading Frame Antigen Network (O.R.A.N.)” to identify immunogenic antigens that are highly likely to act as therapeutic targets. In addition, they created a platform named Tumor-specific Open Reading Frame (TOFU), which employs IVT mRNA technology to encode multiple tumor antigens into a single mRNA vaccine. This allows for the customization of nearly unlimited specific antigens based on each tumor type. mRNA therapies also hold promise for delivering immunomodulators or gene-editing components to spark anti-tumor immune responses in patients or rectify genetic defects. Immunomodulators, such as cytokines, checkpoint inhibitors, and co-stimulatory molecules, can either stimulate or suppress immune responses. Gene-editing components, including Cas9 (CRISPR), effector of transcription activator-like nucleases, and zinc-finger nucleases, can modify the genome of targeted cells. By transporting mRNA coding for these molecules to tumor cells or immune cells, mRNA therapies can provoke tumor cell death, activate immune cells, and modify tumor suppressor genes or oncogenes (113).

### 7.3 mRNA therapies in protein deficiency diseases and rare diseases

Nucleic acid therapies, which regulate protein expression at the genetic level, hold significant potential for treating diseases caused by protein deficiencies or mutations. These approaches offer longer therapeutic half-lives and lower costs compared to protein-based therapies (170, 171). mRNA enters the cell through a delivery system and can translate therapeutic proteins to replace missing or mutated proteins, thereby treating diseases. In cancers with tumor suppressor gene deletions, mRNA can also be used for protein replacement therapy.

Kim et al. (172) screened a peptide, Pep, that specifically binds to PD-L1 and conjugated it to DSPE-PEG2K using copper-free click chemistry, forming DSPE-PEG2K-Pep. This conjugate was incorporated into LNPs to target tumor cells that highly express PD-L1. In triple-negative breast cancer (TNBC), PD-L1 is often overexpressed, and its binding to PD-1 on T cells suppresses their activity and function, allowing tumor cells to evade immune detection. LNP-Pep prevents this by enhancing T cell activity and specifically targeting tumor cells. The LNPs also encapsulate the tumor suppressor gene PTEN, which is frequently deleted or downregulated in TNBC. PTEN suppresses the PI3K-AKT-mTOR signaling pathway, thereby inhibiting tumor cell growth, proliferation, migration, and resistance to apoptosis, and maintaining a normal immune microenvironment. Studies have shown that the mRNA vaccine based on PTEN, delivered via LNPs, can restore PTEN expression, inhibit the PI3K-AKT-mTOR pathway, and demonstrate significant efficacy in the treatment of TNBC.

Rare diseases are characterized by specific genetic mutations, low incidence, severe symptoms, and complex diagnosis and treatment. Many rare diseases are characterized by protein deficiencies. Acute Intermittent Porphyria (AIP) is a rare autosomal dominant metabolic disease caused by a deficiency in porphobilinogen deaminase (PBGD) in the liver. An intravenous injection of PBGD mRNA may take effect within 2 hours, leading to an increase in PBGD expression and enzyme activity in mouse livers. PBGD mRNA also exhibits strong tissue retention therapy capabilities. During mouse experiments, an intravenous injection of human PBGD (hPBGD) mRNA, encoded by the *HMBS* gene and encapsulated in LNPs, induced dose-dependent PBGD protein expression in mouse liver cells. This resulted in the swift clearance of the uroporphyrin precursor. In addition, hPBGD mRNA prevents mitochondrial dysfunction, hypertension, pain, and motor disorders. Repeated administration in AIP mice demonstrated sustained efficacy and therapeutic improvement without evidence of hepatotoxicity. Finally, safety and translatability were confirmed via repeated dosing in non-human primates (173).

Ornithine transcarbamylase deficiency occurs due to the loss of a key enzyme primarily expressed in the liver in the urea cycle, leading to elevated ammonia levels in the blood, which results in neurological damage, coma, and even death. Research in a mouse model of ornithine transcarbamylase deficiency found that delivering mRNA encoding ornithine transcarbamylase with nanoparticles normalized plasma ammonia and urinary lactate levels, extending mouse survival (174).

Phenylketonuria (PKU), also known as phenylalanine hydroxylase (PAH) deficiency, is an autosomal recessive genetic disorder that affects the metabolism of phenylalanine. Mutations in the *PAH* gene lead to significantly elevated levels of phenylalanine in the body, causing abnormal brain function. The primary intervention for PKU is lifelong, strict dietary restriction of phenylalanine, but maintaining this diet is challenging and may still result in neurological complications (175). FDA-approved drugs, Kuvan^®^ and Palynziq^®^, are indicated for specific types of PKU patients, but they do not meet the needs of most patients, highlighting the urgent need for the development of new alternative therapies (176). Cacicedo et al. (177) designed and synthesized LNP-encapsulated mRNA encoding mouse phenylalanine hydroxylase (MmPah mRNA). A single injection of MmPah mRNA-LNPs significantly reduced phenylalanine levels in the serum, liver, and brain of mice within 24 hours, with levels remaining low at 48 hours, but gradually increasing thereafter, indicating that the effect of a single injection was short-lived. However, when mice were repeatedly injected every 5 days for a total of 5 injections, phenylalanine levels in the serum, liver, and brain were significantly reduced to within physiological range 24 hours after each injection. Over the 21-day experimental period, repeated injections did not produce significant adverse effects on the mice’s body weight, liver enzymes, or cytokines. After the 5th injection, phenylalanine levels in the brain remained low, indicating the potential for long-term therapeutic benefits with repeated injections.

## 8 Concluding remarks

mRNA vaccines boast several advantages over traditional vaccines, including high versatility, robust efficacy, quick construction, and easy scalability, making them particularly effective in controlling large-scale outbreaks of infectious diseases. This article reviews the progress of mRNA vaccines in terms of their immunogenicity, capping, UTR selection, ORF optimization, tailing, nucleotide modifications, and delivery systems, as well as their applications in infectious diseases, cancer, protein replacement therapy, and rare diseases. Although BNT162b2 and mRNA-1273 have been successfully approved, and numerous mRNA vaccines for various diseases have entered clinical trials, there are still many areas where mRNA vaccine technology needs improvement. These include:

a. Enhancing the purity of mRNA vaccines by reducing template DNA, IVT reaction by-products, and incomplete mRNA, as well as removing nucleotide analogs from synthetic raw materials, can lower the risk of adverse immunogenic reactions such as allergic responses. Pfizer-BioNTech and Moderna’s COVID-19 mRNA vaccines trigger allergic reactions at rates 2-4 times higher than traditional vaccines (113). This heightened response is thought to be due to antibodies generated against components of the nanoparticle delivery system or the mRNA molecules themselves, which can lead to severe immune reactions, thus triggering inflammation and allergic reactions. Optimizing the composition of the nanocarriers and modifying the mRNA molecules are essential steps in preventing or reducing adverse reactions while enhancing transfection efficiency and stability.

b. Addressing the challenges of mRNA vaccines in transportation and storage as they require cold chain transportation and frozen storage. BNT162b2 can be stored 6 months at -60°C to -80°C, but only 5 days in a 2°C-8°C refrigerator, while mRNA-1273 can be stored for 6 months in a -20°C freezer and a month in a 2°C-8°C refrigerator (34). The specific requirements for transporting and storing mRNA vaccines impose an economic burden, especially in remote and underdeveloped areas. There is a need to develop mRNA vaccines that can be stored and transported at room temperature or even under high-temperature conditions. Notably, freeze-drying technology can significantly extend the storage duration of mRNA vaccines at room temperature, refrigerated, and frozen conditions. However, reconstitution before administration may lead to particle aggregation, which can negatively impact the vaccine’s efficacy. Additionally, the lyophilization process is expensive, labor-intensive, and time-consuming, indicating a need for further improvement.

c. Optimizing mRNA components to enhance stability. Onpattro^®^, an approved siRNA-based gene product, utilizes an LNP delivery system with components similar to those in the two approved COVID-19 mRNA vaccines. Its longevity of 3 years at 2°C-8°C highlights the structure of the mRNA itself (34). In addition to optimizing components, researchers can focus on developing circRNA to further improve mRNA stability and translation efficiency. At the same time, relatively high-dose administration of BNT162b2 (30 µg) and mRNA-1273 (100 µg) vaccines is prone to cause side effects related to innate immune stimulation. Therefore, saRNA and taRNA need to be deeply optimized. Due to their self-replicating properties, they can avoid repeated administration of mRNA vaccines, reduce the side effects of vaccines and increase patient compliance; as a key factor affecting mRNA vaccine stability.

d. Optimizing construct design. Adding signal peptides and endocytic sorting motifs of endosomal or lysosomal transmembrane proteins to the mRNA sequence alters the processing and trafficking of the encoded antigen within host cells, directing it to the endosomal-lysosomal pathway. This enhances the presentation of antigens on MHC-I and MHC-II molecules in dendritic cells, thereby improving immunogenicity (178).

e. Enhancing the research and development of multiple immune adjuvants. mRNA vaccines, utilized in fields such as cancer treatment and infectious diseases, need to stimulate a strong immune response in the body. Therefore, immune adjuvants can be added. Research has shown that adding low doses of the glycolipid alpha-galactosylceramide (αGC) to the C12-200 mRNA vaccine significantly enhances both innate and cellular immune responses. The mechanism involves αGC acting as a glycolipid antigen that binds to specific receptors on invariant natural killer T (iNKT) cells, activating them to secrete a large quantity of cytokines. These cytokines play a key role in recruiting and activating other innate and adaptive immune cells, including dendritic cells, macrophages, natural killer (NK) cells, T cells, and B cells, particularly demonstrating a dose-sparing effect in inducing cellular immune responses. Additionally, iNKT cells exert direct cytotoxic effects, such as targeting and killing infected or tumor cells. Furthermore, iNKT cells influence the microenvironment in infectious diseases and cancer by modulating myeloid cells that have immunosuppressive characteristics (178).

f. Developing targeted mRNA vaccines to precisely target specific tissues or cells, thus avoiding side effects caused by systemic exposure.

g. Optimizing the administration of mRNA vaccines. For pathogens invading through the respiratory or digestive tracts, inhalation or oral administration may be preferable to mimic the way pathogens invade the body, inciting a stronger systemic immune response. Abbasi et al. (179) utilized a liquid jet injector to effectively introduce naked mRNA into skin cells. The skin is rich in APCs, which, upon uptake of the mRNA, migrate to LNs to present the antigen. Additionally, the injection method induces local lymphocyte infiltration in the skin, acting as a physical adjuvant for vaccination. Since no delivery vehicle is used, this approach confines mRNA distribution to the injection site, preventing systemic leakage and associated systemic pro-inflammatory responses. In mouse vaccination studies, naked mRNA delivered via jet injection elicited robust antigen-specific antibody production for over six months, while also promoting the formation of germinal centers in the LNs and the induction of CD4^+^ and CD8^+^ T cells.

h. Enhancing the accessibility of mRNA manufacturing processes. The manufacturing of mRNA vaccines requires standardized and high-quality raw materials, specialized equipment, and facilities for synthesis, purification, and formulation. These requirements present technical and logistical challenges for large-scale production and distribution of mRNA therapies (180).

i. Strengthening the Development of Virus-Like Particle (VLP) mRNA Vaccines. Researchers utilized mRNA to encode the key proteins constituting VLP. Once co-expressed within cells, these proteins self-assemble to form VLP. Several VLP vaccines have already been approved for combating human papillomavirus, hepatitis B virus, and rabies virus. VLPs mimic the structure of natural viruses and feature highly repetitive antigenic epitopes while lacking viral genomes, effectively cross-linking B cell receptors to activate B cells. With sizes smaller than 200 nanometers, VLPs are readily presented by dendritic cells at the injection site, triggering robust adaptive immune responses. Moreover, VLPs extend retention time in lymph node follicles, facilitating presentation by follicular dendritic cells and activating T follicular helper (Tfh) cells and B cells. This enhances germinal center (GC) activation and induces long-lived plasma cells (LLPCs) and memory B cells (MBCs), leading to a sustained humoral response characterized by high titers of neutralizing antibodies (181). Notably, studies have indicated that nucleoside-modified mRNA-LNP vaccines can also induce high levels of Tfh and GC B cells (182).

j. Advancing mRNA vaccine development using pre-fusion stabilized viral surface proteins, a principle that is extendable to other viral vaccines. Some studies have indicated that these pre-fusion forms present antigenic epitopes and induce neutralizing antibodies more effectively than wild-type viral surface proteins and have stronger immunogenicity (181).

In summary, despite a need for continued refinement, mRNA vaccines’ demonstrated advantages in infectious diseases, cancer, protein replacement therapy, and prevention and treatment of rare diseases indicate significant potential for application.
